# Analysis of the causes and clinical characteristics of jejunoileal hemorrhage in China: a multicenter 10 year retrospective survey

**DOI:** 10.1186/1471-230X-12-101

**Published:** 2012-08-06

**Authors:** Da-lei Jiang, Hui-ya Liu, Yong Yuan, Jian-chao Sui, Chang-chun Jing, Kai-tong Jiang, Qing-cai Wang, Sheng-an Yuan, Hai-ying Chen, Yan-jing Gao

**Affiliations:** 1 Department of Gastroenterology, Qilu Hospital of Shandong University, Jinan, China; 2 The Medical Department The Affiliated Hospital of Medical College, Qingdao University, Qingdao, China; 3 Department of Gastroenterology, Wen-Deng People's Hospital, Wen-Deng, China; 4 Department of Gastroenterology, Liao-cheng Second People's Hospital, Liao-cheng, China; 5 Department of Gastroenterology, Lin-Yi People's Hospital, Lin-Yi, China; 6 Department of Gastroenterology, Tai-An People Hospital, Tai-An, China; 7 Department of Gastroenterology, Zi-bo People's Hospital, Zi-bo, China; 8 Department of Gastroenterology, Qing-Dao Municipal Hospital, Qing-Dao, China; 9 Department of Gastroenterology, He-Ze Municipal Hospital, He-Ze, China

**Keywords:** Cause, Clinical features, Diagnosis, Jejunoileal hemorrhage

## Abstract

**Background:**

A retrospective study was performed to assess the causes, diagnostic methods for, and clinical features of, jejunoileal hemorrhage in Shandong province, China and to derive recommendations for management of this condition from these data.

**Methods:**

We performed a retrospective systematic collection of data from between January 1999 and December 2008 in seven cities in Shandong province, China, identified 72 patients with jejunoileal hemorrhage and analyzed the relevant clinical data.

**Results:**

Overall, tumors were the most common cause of jejunoileal hemorrhage (42 patients, 58.3%). The causes of this condition were significantly different (*P* < 0.05) in male and female patients. In male patients, the commonest factors were tumor (52.2%), enteritis (17.4%) and angiopathy (15.2%). However, in female patients, tumors accounted for a greater proportion of cases (18/26, 69.2%). In 38 cases (52.8%) the diagnosis was made by intraoperative enteroscopy or laparotomy, in 14 by capsule endoscopy and in the remainder by radiological methods. The most frequent presentation was melena (62.7%), followed by maroon stools (26.9%) and hematochezia (9.0%). Of the 72 patients,laparotomy is the main treatment method.

**Conclusion:**

Tumor, enteritis and angiopathy and diverticular disease are the most common causes of jejunoileal hemorrhage in Shandong province, China. The main clinical manifestations are bloody stools, most commonly in the form of melena, with or without abdominal pain. We recommend that female patients over the age of 40 with jejunoileal hemorrhage accompanied by abdominal pain should undergo urgent further assessment because of the strong probability of jejunoileal tumor.

## Background

Jejunoileal hemorrhage is bleeding from anywhere between the ligament of Treitz and the ileocecal valve: the jejunum and ileum. Obscure gastrointestinal bleeding (OGIB) is blood loss from an unknown source that persists or recurs after negative investigations including esophagogastroduodenoscopy (EGD), colonoscopy and radiologic evaluation [1]. OGIB is the initial classification in about 5% of all patients with bleeding from the digestive tract [2]. OGIB originates most commonly from the small intestine; the jejunoileum is reportedly subsequently identified as the origin of bleeding in 75% of patients initially classified as having OGIB [1,3-5]. Patients with jejunoileal hemorrhage usually undergo multiple diagnostic procedures and blood transfusions and are thus a heavy burden on healthcare resources.

In recent years, there have been many advances in the diagnosis and treatment of bleeding from the small bowel, these include capsule endoscopy and push endoscopy. However, especially in China, few studies have focused on the clinical features and the correlations between causes and clinical manifestations in patients with jejunoileal hemorrhage [1,2,6-15]. In the Western world**,** the most common diagnoses in patients with gastrointestinal hemorrhage suspected to be from the small bowel are vascular lesions such as angiodysplasia, followed by erosions and ulcerations of various causes, and then small-bowel tumors [6-8]. Another study showed that the likely cause of small intestinal bleeding depends on the patient’s age. Younger patients are more likely to have small intestinal tumor, Meckel’s diverticulum, Dieulafoy’s lesion, or Crohn’s disease, whereas vascular lesions comprise up to 40% of all causes in patients aged over 40 years, followed by nonsteroidal anti-inflammatory drug–induced small bowel disease [1].

How much of the above is true in China? This is difficult to ascertain because jejunoileal bleeding is not easy to diagnose early: its symptoms are nonspecific and some hospitals have few diagnostic techniques available. Therefore, we retrospectively reviewed and analyzed 72 patients who were admitted to hospitals for jejunoileal bleeding in seven cites in Shandong province, China from January 1999 to December 2008. We present here a detailed investigation and discussion of the causes and clinical features of jejunoileal hemorrhage in this cohort, in order to provide a more complete and up-to-date picture of this condition in China.

### Patients and methods

We studied 72 patients from seven cities in Shandong province, China who had been admitted to hospitals for jejunoileal bleeding from January 1999 to December 2008. The Ethics Committee of Shandong University Qi-Lu Hospital approved our research protocol and the study followed standard ethical guidelines. The admission criterion was a first diagnosis of jejunoileal hemorrhage made during a patient’s admission. We collected the following data for each patient and entered it on a standardized form: admission number, name, age, sex, course of disease, initial symptoms, clinical manifestations, amount of blood loss, color of bloody stools, cause, means of diagnosis, complications, surgery undertaken and time between initial presentation and diagnosis.

The participating centers and number of cases for each center were as follows: Qi-Lu Hospital of Shandong University, Jinan city (31 cases); Wendeng Central People's Hospital, Wendeng city (14 cases); Second Liaocheng People's Hospital, Liaocheng city (8 cases); Linyi People's Hospital, Linyi city (7 cases); Qingdao Municipal Hospital, Qingdao city (4 cases); Zibo Central People's Hospital, Zibo city (4 cases); and Taian Central People’s Hospital, Taian city (4 cases). There were 46 male and 26 female patients of mean age 47 years. The mean duration of disease was 390 days.

After excluding two patients with severe abdominal pain, we enrolled 70 of the 72 eligible patients for analysis. The enrolled patients had all undergone gastroduodenoscopies and colonoscopies and excluded lesions of the stomach, duodenum, colon and rectum.

### Statistical analysis

We compared differences between means using SPSS11.5 statistical software. We performed intergroup analyses with the χ^2^ test. *P <* 0.05 was considered significant.

## Results

### Relevant clinical data and causes of jejunoileal bleeding

Of the 72 patients in the study, 46 (63.9%) were male and 26 (36.1%) female (ratio 1.8:1).Their mean age was 47 years (range 13–85 years). 76.4% of patients were aged over 40 years. The mean age of the patients with small bowel tumors was 53 years. In 35 cases, the condition had been present for less than 1 month, in 21 cases for between 1 month and 1 year, and in 16 cases for more than 1 year. The longest duration was 10 years. The amount of blood lost varied between 50 mL and 2000 mL.

We have listed relevant clinical variables and causative factors for the 72 patients in Table 1. We observed tumors, the leading cause of jejunoileal hemorrhage in our study, in 42 patients (58.3%). We found the second most frequent factor, enteritis or subepithelial ulcerated lesions, in 9 patients (12.5%).And angiopathy and diverticula of the jejunoileal region were found in seven patients (9.7%), respectively. We classified four cases (5.6%) as miscellaneous (including one with small intestinal duplicationone, one with Purpura, one with drug-induced intestinal bleeding and one case of ectopic pancreas) and in three (4.2%) the cause was Crohn^’^s disease.

**Table 1:** Demographic features and causes in patients with jejunoileal hemorrhage in 7 cities of China

**No. (%)**		**Sex**		**Age**		**Mean course of disease(d)**	**No. of Abdominal pain**
		**Male**^a^**(%)**	**Female**^a^**(%)**	**≤40**	**>40**		
Tumor^b^	42(58.3)	24(52.2)	18(69.2)	5	37	283	16
Enteritis	9(12.5)	8(17.4)	1(3.8)	3	6	620	4
Angiopathy	7(9.7)	7(15.2)	0(0.0)	2	5	204	0
Diverticulum	7(9.7)	5(10.9)	2(7.7)	3	4	100	3
Crhon’s disease	3(4.2)	1(2.2)	2(7.7)	3	0	133	2
Miscellaneous^c^	4(5.6)	1(2.2)	3(11.5)	1	3	451	1
Total	72	46	26	17	55		26

Note:^a^Deference in constituent ratio of cause of jejunoileal hemorrhage between male and female, *P*<0.05.

^b^Tumors: including Hemangioma, leiomyoma, adenoma, stromal tumor, adenocarcinoma, desmoplastic small round cell tumor, lymphoma, malignant peripheral nerve sheath tumors, spindle cell tumor - myofibroblastic tumor.

^c^Miscellaneous: including one with small intestinal duplicationone, one with Purpura, one with drug-induced intestinal bleeding and one case of ectopic pancreas.

There was a significant difference between male and female patients in the ratio of the various causes of jejunoileal hemorrhage (*P* < 0.05). The most frequent factor in men was tumors (24/46, 52.2%), followed by enteritis or subepithelial ulcerated lesions (8/46, 17.4%), angiopathy (7/46, 15.2%), diverticula (5/46, 10.9%) and Crohn^’^s disease (1/46, 2.2%). In the female patients, tumors were also the main causative factor (18/26, 69.2%). Diverticula were the cause in 2/26 female patients (7.7%) and Crohn^’^s disease in 2/26 (7.7%). However, we found enteritis in only 1/26 patients (3.8%), and angiopathy in none of the 26 female patients.

There was also a significant difference between patients older and younger than 40 years in the cause of jejunoileal hemorrhage (*P* < 0.05). Among the 55 patients who were older than 40 years, the most frequent cause was tumors (37/55, 67.3%), followed by enteritis or subepithelial ulcerated lesions (6/55, 11%), angiopathy (5/55, 9.1%), and diverticula (4/55, 7.3%). Among the 17 patients aged 40 years or younger, the most frequent cause was also tumors (5/17, 29.4%), followed by enteritis or subepithelial ulcerated lesions(3/17, 17.7%), diverticula(2/17, 11.8%) and Crohn^’^s disease (3/17, 17.7%) and angiopathy (2/17, 11.8%).

**Figure 1:**
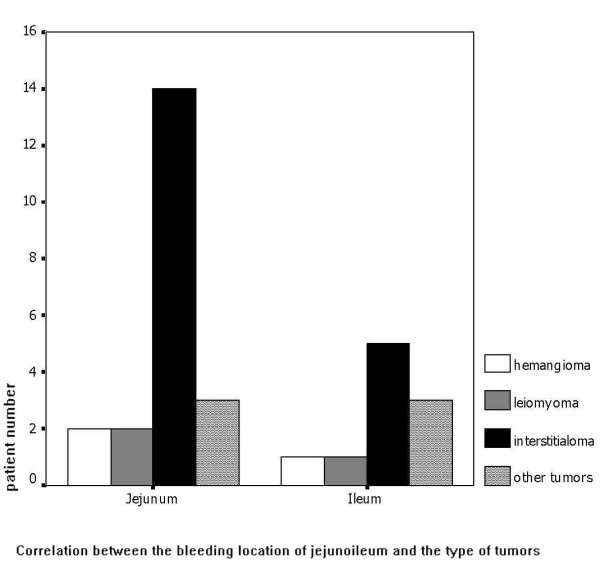
Correlation between the bleeding location of jejunoileum and the type of tumors.

We defined the location of bleeding and diagnosed the type of tumor in 31 of the 42 patients with jejunoileal tumors. In 21 cases (67.7%), the bleeding was from the jejunum and in 10 cases (32.3%) from the ileum (Figure 1). Stromal tumors and adenocarcinomas were the two most frequent tumor types for both jejunal and ileal bleeding.

### Initial symptoms and main clinical manifestations

The commonest initial symptom of bloody stools occurred in 67 patients, in 23 this was accompanied by abdominal pain. Other initial symptoms were abdominal pain accompanied by fecal occult blood (one patient) and severe abdominal pain without hematochezia (two patients). In two patients who presented with severe abdominal pain associated with shock, abdominocentesis and subsequent exploratory laparotomy revealed non-coagulated bloody liquid and frank blood in the abdominal cavity or within the small intestines. As to the appearance of the bleeding, 42 patients (42/72, 58.3%) had melena, followed by maroon stools in 19 (19/72, 26.4%), red stools in 6 (6/72, 8.3%) and no visible fecal blood (occult blood detected by testing) in 1 (1.4%). We estimated that 57 patients lost less than 800 mL the other 15 patients more than or equal to 800 mL of blood.

We found tumors in 16/26 patients with abdominal pain. 12 of these patients had malignant tumors and the four had benign tumors. Other causes of abdominal pain included enteritis (four cases), diverticula (three cases), Crohn’s disease (three cases) .The patients with angiopathy had no abdominal pain.

We located the source of the bleeding in 54/72 patients. It was jejunal in 23 and ileal in 31 patients (Table 2). Jejunal bleeding was most often caused by tumors (21/23, 91.3%), the other two patients had angiopathy (one) and diverticula (one). Tumors also ranked first (12/31, 38.7%) in the patients with ileal bleeding, followed by enteritis (6/31, 19.4%), angiopathy (5/31, 16.1%), diverticula (5/31, 16.1%) , Crohn^’^s disease (1/31, 3.2%) and miscellaneous(2/31, 6.5%). Thus, there was a significant difference between jejunal and ileal bleeding (*P* < 0.05) in the ratio of the various causes of hemorrhage. The appearance of the stools also differed. Patients bleeding from the jejunum had melena, maroon and red stools in 15/23 (68.2%), 7/23 (31.8%) and zero cases, respectively; Patients bleeding from the ileum had melena, maroon and red stools in 17/31 (60.7%), 8/31 (28.6%) and 3/31 (28.7%), respectively.

**Table 2:** Correlation among the causes, location of jejunoileal hemorrhage and bleeding in different degrees

**No.**		**Location**	
		**Jejunum**^a^	**Ileum**^a^
Tumor	33	21	12
Enteritis	6	0	6
Angiopathy	6	1	5
Diverticulum	6	1	5
Crhon’s disease	1	0	1
Miscellaneous^b^	2	0	2
Total	54	23	31

Note:^a^ There is significant deference in the constituent ratio of cause of jejunoileal hemorrhage between Jejunum and ileum bleeding *P*<0.05.

^b^Miscellaneous: including one with small intestinal duplicationone and one case of ectopic pancreas.

### Frequency of hospitalization, complications and treatment modalities

Of the 72 patients, 37 cases (51.4%) were diagnosed during their first admission, 18 (25.0%) on their second admission, and 17 (23.6%) on their third. Thirty patients had various complications including anemia in 16/30 cases (53.3%), intestinal obstruction in 6/30 (20%), abdominal metastases in 3/30 (10.0%), shock in 2/30 (6.7%), intestinal perforation in 2/30 (6.7%), and ankylenteron in 1/30 (3.3%) (Table 3). Tumors were most frequent causes of these complications, accounting for 66.7% (20/30). Fifty of the patients were treated surgically and the remaining 22 by conservative medical means.

**Table 3:** Relation between causes of jejunoileal bleeding and complication

	**Complication**					
	**Anemia**	**Intestinal obstruction**	**Enteric perforation**	**intestinal adhesion**	**Abdominal visceral metastasis**	**Shock**
Tumor	7	6	1	1	3	2
Enteritis	1	0	1	0	0	0
Angiopathy	3	0	0	0	0	0
Diverticulum	4	0	0	0	0	0
Crhon’s disease	0	0	0	0	0	0
Miscellaneous^a^	1	0	0	0	0	0
Total	16	6	2	1	3	2

Miscellaneous^a^: One case of drug-induced intestinal bleeding.

### Diagnostic procedures

The diagnosis was made by intraoperative endoscopy or laparotomy in 38 of the 72 patients. Their diagnoses were tumors in 28 cases, diverticula in 5, enteritis in 4 and Crohn’s disease in 1. Capsule endoscopy (CE) examination definitively identified the source of bleeding in 17/25 patients (68%). Seven of 14 patients (50%) who underwent selective arteriography were found to have jejunoileal bleeding. Jejunoileal lesions were identified in 5/53 patients (9.4%) who underwent barium and air double contrast X-ray examinations. The details are presented in Table 4.

**Table 4:** No. of cases diagnosed by various examination procedures

	**Tumor**	**Enteritis**	**Angiopathy**	**Diverticulum**	**Crhon’s disease**	**Miscellaneous**	**Total(n)**
CE	7	2	5	1	0	2	17
Enteroscopy	2	0	0	0	0	0	2
Air barium double contrast X-ray	1	0	0	4	0	0	5
Selective arteriography	4	0	2	0	0	1	7
Intraoperative endoscopy and laparotomy	28	4	0	5	1	1	39
Colonoscopy	0	0	0	0	2	0	0

Note: In 4 cases of Miscellaneous, One case with Purpura and one with drug-induced intestinal bleeding were diagnosed by CE; One case with duplicated deformity of the ileum was diagnosed by selective arteriography; One case of ectopic pancreas was diagnosed by laparotomy and pathology.

## Discussion

In the United States and Europe, the causes of small bowel bleeding have been well established; vascular abnormalities are the commonest cause with a frequency of 30-40% [16,17]. Tumors reportedly account for only about 6.4% of small intestinal bleeding in these regions [16]. However, in our series of Chinese patients tumors (58.3%) were the most frequent cause of small bowel bleeding, followed by enteritis or subepithelial ulcerated lesions (12.5%), diverticula of the small intestine (9.7%), angiopathy (9.7%), Crohn’s disease (4.2%) and other diseases (5.6%). Thus, there are major differences between western countries and China in the causes of small bowel bleeding. We found that tumors were the leading cause of jejunoileal bleeding both in patients older and younger than 40 years; however, the proportion of jejunoileal hemorrhage caused by tumors did differ significantly between these age groups (*P* < 0.05). The reasons for this difference are unknown and may include inherited factors.

Benign tumors are reportedly more often responsible for small intestinal bleeding than malignant tumors. The most common benign tumor that causes small bowel bleeding is leiomyoma, whereas the commonest malignant tumor is leiomyosarcoma. Benign tumors seem to bleed more often than malignant tumors [18]. In contrast, malignant tumors (which were most often primary interstitialomas) accounted for 75% of all the tumor-related jejunoileal bleeding in our series. Benign tumors (which were mainly primary angiomas and leiomyomas) accounted for only 25%. In the 33 cases with tumors and identified location of bleeding, 63.6% (21/33) of those tumors were located in the jejunum. However, bleeding caused by enteritis, diverticula and angiopathy of the small intestine most often originated in the ileum. The difference between causes of hemorrhage from the jejunum versus from the ileum was significant (*P* < 0.05).

In our series, repeated, intermittent hematochezia or hematochezia accompanied by abdominal pain were the commonest initial symptoms of patients with jejunoileal bleeding. This most frequently took the form of melena (58.3%), followed by maroon (25.0%) and red stools (8.3%). There was a statistically significant difference (*P* < 0.05) between male and female patients in the cause of jejunoileal hemorrhage. Tumors, followed by enteritis or subepithelial ulcerated lesions and angiopathy, were the most frequent cause in men. However in the 26 women, although tumors were also the main cause (18/26, 69.2%), no case of angiopathy were identified. We recommend that female patients aged over 40 years with hematochezia accompanied by abdominal pain and a high suspicion of a small bowel bleeding should undergo urgent further examination because of the high probability of jejunoileal tumor. For male patients with copious jejunoileal bleeding that is not accompanied by abdominal pain, we suggest that the diagnosis of angiopathy should be considered after jejunoileal tumor has been excluded.

Current options for the diagnosis and management of small bowel bleeding include selective angiography, radiographic means such as small bowel series and enteroclysis, push enteroscopy, CE, single-balloon enteroscopy, double-balloon enteroscopy, intraoperative enteroscopy (IOE), and exploratory laparotomy [15,19-25]. Because it is a noninvasive procedure, capsule endoscopy is recommended as the first diagnostic test for patients with suspected small bowel bleeding in developed countries [10,17]. IOE and exploratory laparotomy are now accepted as the procedure of choice for complete evaluation of the small bowel [24,25]. However, in our series only 54.2% of all cases were diagnosed by IOE and laparotomy, followed by 23.6% by capsule endoscopy, 9.7% by selective angiography, 8.3% by small bowel series and enteroclysis and 2.8% by both push enteroscopy and colonoscopy. A diagnosis was made during the first hospital admission in only 51.4% of all cases in our series. Conversely, a diagnosis was not made during the first admission in about half of the patients in our series. This may be because China is still a developing country and CE examination was unavailable in many of the surveyed hospitals in the years we studied, especially before 2005.

All of the 72 patients with jejunoileal hemorrhage had obtained a surgical consultation. And according to the surgeon's advice, 50 patients underwent surgical treatment including 42 of tumors,7 of angiopathy and 1 of enteritis. The remaining 22 were treated by conservative medical means. Surgical treatment was indicated so often in these patients because tumors are the major cause of jejunoileal bleeding in China.

In our series, 41.7% of cases had various complications including anemia, intestinal obstruction, abdominal metastasis, shock, ankylenteron, and intestinal perforation. The commonest causes of complications were tumors; 47.6% of the complications were in tumor patients. Conversely, 66.7% of patients with tumors had complications. Anemia, intestinal obstruction, abdominal metastases, shock, ankylenteron and intestinal perforation were all primarily found in tumor patients. Thus, patients presenting with jejunoileal bleeding and serious complications have a high probability of having small bowel tumors.

## Conclusions

The results of this study clearly show a more update view of the the causes, diagnostic methods and clinical features of jejunoileal hemorrhage in China. Tumor, enteritis and angiopathy and diverticular disease are the most common causes of jejunoileal hemorrhage in Shandong province, China. The main clinical manifestations are bloody stools, most commonly in the form of melena, with or without abdominal pain. We recommend that female patients over the age of 40 with jejunoileal hemorrhage accompanied by abdominal pain should undergo urgent further assessment because of the strong probability of jejunoileal tumor. For male patients with copious jejunoileal bleeding that is not accompanied by abdominal pain, we suggest that the diagnosis of angiopathy should be considered after jejunoileal tumor has been excluded. Laparotomy is the main treatment method, based on the data that indicate that the main cause is tumor.

## Abbreviations

OGIB, Obscure Gastrointestinal Bleeding; EGD, EsophagoGastroduoDenoscopy; CE, Capsule Endoscopy.

## Competing interests

None of the authors has any financial or non-financial competing interests influencing interpretation of data or presentation of information.

## Authors' contributions

DJ and HL contributed equally to this work. YG is the corresponding author. D J: Selected patients,carried out the study and drafted the manuscript. HL: Selected patients,carried out the study. YY: Design the study and carried out the statistical analysis.YG: Design the study and drafted the manuscript. JS: Selected patients and carried out the study. CJ: Selected patients and carried out the study. KJ: Selected patients and carried out the study. QW: Selected patients and carried out the study. SY: Selected patients and carried out the study. HC: Selected patients and carried out the study. All authors read and approved the final manuscript.

## Pre-publication history

The pre-publication history for this paper can be accessed here:

http://www.biomedcentral.com/1471-230X/12/101/prepub

## References

[B1] Raju GS Gerson L Das A Lewis BS American Gastroenterological, Association (AGA) Institute technical review on obscure gastrointestinal bleeding Gastroenterology 2007 133 1697 1717 17983812 10.1053/j.gastro.2007.06.007

[B2] Almeida N Figueiredo P Lopes S Freire P Lérias C Gouveia H Leitão MC Urgent capsule endoscopy is useful in severe obscure-overt gastrointestinal bleeding Dig Endosc 2009 21 87 92 19691780 10.1111/j.1443-1661.2009.00838.x

[B3] Rockey DC Occult gastrointestinal bleeding N Engl J Med 1999 341 38 46 10387941 10.1056/NEJM199907013410107

[B4] American Gastroenterological Association medical position statement Evaluation and management of occult and obscure gastrointestinal bleeding Gastroenterology 2000 118 197 201 10611169 10.1016/s0016-5085(00)70429-x

[B5] Gralnek IM Obscure-overt gastrointestinal bleeding Gastroenterology 2005 128 1424 1430 15887123 10.1053/j.gastro.2005.03.067

[B6] May A Nachbar L Ell C Double-balloon enteroscopy (push-and-pull enteroscopy) of the small bowel: feasibility and diagnostic and therapeutic yield in patients with suspected small bowel disease Gastrointest Endosc 2005 62 62 70 15990821 10.1016/s0016-5107(05)01586-5

[B7] Heine GD Hadithi M Groenen MJ Kuipers EJ Jacobs MA Mulder CJ Double-balloon enteroscopy: indications, diagnostic yield, and complications in a series of 275 patients with suspected small-bowel disease Endoscopy 2006 38 42 48 16429354 10.1055/s-2005-921188

[B8] Hadithi M Heine GD Jacobs MA van Bodegraven AA Mulder CJ Eickhoff A Huschner W Möller K Jakobs R Reitzig P Weickert U Gellert K Schultz H Guenther K Hollerbuhl H Schoenleben K Schulz HJ Riemann JF A prospective study comparing video capsule endoscopy with double-balloon enteroscopy in patients with obscure gastrointestinal bleeding Am J Gastroenterol 2006 101 52 57 16405533 10.1111/j.1572-0241.2005.00346.x

[B9] Waye JD Small-intestinal endoscopy Endoscopy 2001 33 24 30 11204984 10.1055/s-2001-11184

[B10] Nakamura T Terano A Capsule endoscopy: past, present, and future J Gastroenterol 2008 43 93 9 Pubmed: 18306982 10.1007/s00535-007-2153-6 18306982

[B11] Mönkemüller K Neumann H Meyer F Kuhn R Malfertheiner P Fry LC A retrospective analysis of emergency double-balloon enteroscopy for small-bowel bleeding Endoscopy 2009 41 715 717 19670141 10.1055/s-0029-1214974

[B12] Marmo R Rotondano G Casetti T Manes G Chilovi F Sprujevnik T Bianco MA Brancaccio ML Imbesi V Benvenuti S Pennazio M Degree of concordance between double-balloon enteroscopy and capsule endoscopy in obscure gastrointestinal bleeding: a multicenter study Endoscopy 2009 41 587 592 19588285 10.1055/s-0029-1214896

[B13] Jakobs R Hartmann D Benz C Schilling D Weickert U Eickhoff A Schoenleben K Riemann JF Diagnosis of obscure gastrointestinal bleeding by intra-operative enteroscopy in 81 consecutive patients World J Gastroenterol 2006 12 313 316 16482636 10.3748/wjg.v12.i2.313 PMC4066045

[B14] Cobrin GM Pittman RH Lewis BS Increased Diagnostic Yield of Small Bowel Tumors With Capsule Endoscopy Cancer 2006 107 22 27 16736516 10.1002/cncr.21975

[B15] Shinozaki S Yamamoto H Yano T Sunada K Miyata T Hayashi Y Arashiro M Sugano K Long-term Outcome of Patients With Obscure Gastrointestinal Bleeding Investigated by Double-Balloon Endoscopy Clin Gastroenterol Hepatol 2010 8 151 158 19879968 10.1016/j.cgh.2009.10.023

[B16] Hartmann D Schmidt H Bolz G A prospective two-center study comparing wireless capsule endoscopy with intraoperative enteroscopy in patients with obscure GI bleeding Gastrointest Endosc 2005 61 826 832 15933683 10.1016/s0016-5107(05)00372-x

[B17] Gerson LB Capsule Endoscopy and Deep Enteroscopy: Indications for the Practicing Clinician Gastroenterology 2009 137 1197 1201 19703454 10.1053/j.gastro.2009.08.035

[B18] Rockey DC Occult and obscure gastrointestinal bleeding: causes and clinical management Nat Rev Gastroenterol Hepatol 2010 7 265 279 20351759 10.1038/nrgastro.2010.42

[B19] Triester SL Leighton JA Leontiadis GI Gurudu SR Fleischer DE Hara AK Heigh RI Shiff AD Sharma VK A meta-analysis of the yield of capsule endoscopy compared to other diagnostic modalities in patients with non-stricturing small bowel Crohn’s disease Am J Gastroenterol 2006 101 954 964 16696781 10.1111/j.1572-0241.2006.00506.x

[B20] Triester SL Leighton JA Leontiadis GI Fleischer DE Hara AK Heigh RI Shiff AD Sharma VK A meta-analysis of the yield of capsule endoscopy compared to other diagnostic modalities in patients with obscure gastrointestinal bleeding Am J Gastroenterol 2005 100 2407 2418 16279893 10.1111/j.1572-0241.2005.00274.x

[B21] May A Färber M Aschmoneit I Pohl J Manner H Lotterer E Möschler O Kunz J Gossner L Mönkemüller K Ell C Prospective multicenter trial comparing push-and-pull enteroscopy with the single- and double-balloon techniques in patients with small-bowel disorders Am J Gastroenterol 2010 105 575 581 20051942 10.1038/ajg.2009.712

[B22] Ramchandani M Reddy DN Gupta R Lakhtakia S Tandan M Rao GV Darisetty S Diagnostic yield and therapeutic impact of single-balloon enteroscopy: series of 106 cases J Gastroenterol Hepatol 2009 24 1631 1638 19686408 10.1111/j.1440-1746.2009.05936.x

[B23] Kendrick ML Buttar NS Anderson MA Lutzke LS Peia D Wang KK Sarr MG Contribution of intraoperative enteroscopy in the management of obscure gastrointestinal bleeding J Gastrointest Surg 2001 5 162 167 11331479 10.1016/s1091-255x(01)80029-9

[B24] Hartmann D Schmidt H Bolz G Schilling D Kinzel F Eickhoff A Huschner W Möller K Jakobs R Reitzig P Weickert U Gellert K Schultz H Guenther K Hollerbuhl H Schoenleben K Schulz HJ Riemann JF A prospective two-center study comparing wireless capsule endoscopy with intraoperative enteroscopy in patients with obscure GI bleeding Gastrointest Endosc 2005 61 826 832 15933683 10.1016/s0016-5107(05)00372-x

[B25] Schulz HJ Schmidt H Intraoperative enteroscopy Gastrointest Endosc Clin N Am 2009 19 371 379 19647646 10.1016/j.giec.2009.04.011

